# Synthetic Oligodeoxynucleotides Containing Multiple Telemeric TTAGGG Motifs Suppress Inflammasome Activity in Macrophages Subjected to Oxygen and Glucose Deprivation and Reduce Ischemic Brain Injury in Stroke-Prone Spontaneously Hypertensive Rats

**DOI:** 10.1371/journal.pone.0140772

**Published:** 2015-10-16

**Authors:** Jing Zhao, Yongshan Mou, Joshua D. Bernstock, Dace Klimanis, Sixian Wang, Maria Spatz, Dragan Maric, Kory Johnson, Dennis M. Klinman, Xiaohong Li, Xinhui Li, John M. Hallenbeck

**Affiliations:** 1 Department of Neurology, Jinan Central Hospital affiliated with Shandong University, 105 Jiefang Road, Jinan, Shandong, 250013, P. R. China; 2 Stroke Branch, National Institute of Neurological Disorders and Stroke, National Institutes of Health, Bethesda, Maryland, United States of America; 3 College of Arts and Sciences, Cornell University, Ithaca, New York, United States of America; 4 National Institute of Neurological Disorders and Stroke, Flow Cytometry Core Facility, Bethesda, Maryland, United States of America; 5 Information Technology & Bioinformatics Program, National Institute of Neurological Disorders and Stroke, National Institutes of Health, Bethesda, Maryland, United States of America; 6 Cancer and Inflammation Program, National Cancer Institute, National Institutes of Health, Bethesda, Maryland, United States of America; Chang-Gung University, TAIWAN

## Abstract

The immune system plays a fundamental role in both the development and pathobiology of stroke. Inflammasomes are multiprotein complexes that have come to be recognized as critical players in the inflammation that ultimately contributes to stroke severity. Inflammasomes recognize microbial and host-derived danger signals and activate caspase-1, which in turn controls the production of the pro-inflammatory cytokine IL-1β. We have shown that A151, a synthetic oligodeoxynucleotide containing multiple telemeric TTAGGG motifs, reduces IL-1β production by activated bone marrow derived macrophages that have been subjected to oxygen-glucose deprivation and LPS stimulation. Further, we demonstrate that A151 reduces the maturation of caspase-1 and IL-1β, the levels of both the iNOS and NLRP3 proteins, and the depolarization of mitochondrial membrane potential within such cells. In addition, we have demonstrated that A151 reduces ischemic brain damage and NLRP3 mRNA levels in SHR-SP rats that have undergone permanent middle cerebral artery occlusion. These findings clearly suggest that the modulation of inflammasome activity via A151 may contribute to a reduction in pro-inflammatory cytokine production by macrophages subjected to conditions that model brain ischemia and modulate ischemic brain damage in an animal model of stroke. Therefore, modulation of ischemic pathobiology by A151 may have a role in the development of novel stroke prevention and therapeutic strategies.

## Introduction

Stroke is the second most common cause of death and the third most common cause of disability-adjusted life-years (DALYs) worldwide. Of note, the global burden of stroke as measured by the number of people affected every year, stroke survivors, related deaths, and DALYs lost continues to increase [1]. The immune system plays a critical role in the development and subsequent pathobiology of stroke. Inflammation and immunity have been linked to multiple risk factors for stroke which include hypertension [2], atherosclerosis [3], diabetes [4,5], atrial fibrillation [6], and tobacco smoke-induced vascular impairment [7]. Of particular interest are the damage-associated molecular pattern molecules (DAMPs) released as a result of a cerebrovascular accident, that promote innate immune responses that contribute to brain damage and ultimately to neurological deficits [8,9].

Although therapies directed at the early restoration of perfusion (i.e. recombinant tissue plasminogen activator) have shown clear efficacy within the clinic [10], decades of research focusing on putative mechanisms of cytoprotection that may permit brain cells to maintain homeostasis both during/after an ischemic stress have uniformly failed to translate into clinically relevant therapies [11]. Nonetheless, the basic and translational efforts of the international stroke community have massively advanced the understanding of the governing dynamics underlying stroke pathobiology.

Inflammasomes are multi-protein complexes activated as part of the innate immune response to stressors and/or infections that trigger the maturation of caspase-1 followed by the production of IL-1β and IL-18 [12,13]. Caspase-1 and IL-1β promote inflammation and cell death with IL-1β having been implicated in a number of disease processes including those that unfold after an ischemic injury [14–16]. In experimental stroke, IL-1β expression increases following brain ischemia and multiple studies have shown that blocking IL-1β can be neuroprotective [17]. Of note, in humans IL-1β levels increase in both the cerebrospinal fluid and blood after an ischemic stroke [18–20]. In murine models, levels of inflammasome related proteins increase after an ischemic brain injury and the inhibition of inflammasome activity has been shown capable of reducing the extent of such injuries [21–25].

Telomeres cap the ends of linear chromosomes, protecting them from fusion, degradation, and/or recombination [26]. Mammalian telomeres are composed of repetitive TTAGGG motifs [27]. These motifs are released from dying host cells and serve to down-regulate inflammatory responses that can cause tissue destruction (e.g. as in autoimmune disease) [28,29]. The synthetic oligodeoxynucleotide A151 is composed of four TTAGGG motifs on a phosphorothioate backbone. A151 duplicates the ability of telomeric DNA to modulate inflammation, including the production of IL-6, IL-12, IFNγ, MIP-2, and TNFα [29–31]. A151’s potential as an anti-inflammatory agent has been demonstrated in animal models of arthritis [32], endotoxic shock [31], concanavalin A induced hepatitis [33], ocular inflammation [34], lupus nephritis [35], atherosclerosis [36], and silica-induced pulmonary inflammation [37]. Critically, the pharmacokinetics, pharmacodynamics and safety of phosphorothioate oligodeoxynucleotides have been established in multiple clinical trials [38–40].

Given the role of IL-1β in the development/progression of ischemic brain injury and the success of A151 in ameliorating multiple diseases with inflammatory components, we sought to examine the effect of A151 on critical processes (i.e. inflammasome mediated responses) underlying the pathophysiology of brain ischemia via the synergistic use of both oxygen-glucose deprivation (OGD) and lipopolysaccharide (LPS) stimulation in a reductionist *in vitro* system which is intended to replicate the complex pathobiology that unfolds during an ischemic stroke. We then sought to confirm the potential therapeutic utility of this compound *in vivo* via a murine model of stroke. Per the aforementioned, bone marrow derived macrophages (BMDM) were selected for study because macrophages/microglia are the major sources of IL-1β within the ischemic brain [17]. In addition to perivascular macrophages, monocytes rapidly infiltrate the brain and become macrophages after brain ischemia [8,41]. The effect of A151 on the development of ischemic lesions in stroke-prone spontaneously hypertensive (SHR-SP) rats was investigated by means of permanent middle cerebral artery occlusion (pMCAO), which is a widely accepted animal model of ischemic stroke.

In the course of these studies we found that A151 reduces the maturation of caspase-1 and IL-1β, the production of the inflammasome sensor protein NLRP3, and iNOS, and the depolarization of mitochondrial membrane potential. Further, A151 was also shown to reduce ischemic brain injury (via a decrease in lesional volume) and NLRP3 mRNA in SHR-SP rats. Therefore, this type of anti-inflammatory agent may thus represent a novel approach to the prevention and/or treatment of ischemic stroke.

## Materials and Methods

### Reagents

Phosphorothioate oligodeoxynucleotides (ODN) A151 (5’-TTAGGGTTAGGGTTAGGGTTAGGG-3’) and control ODN C151 (5’-TTCAAATTCAAATTCAAATTCAAA-3’) were synthesized at the FDA CBER Core Facility (Silver Spring, MD). These ODNs were free of both detectable protein and endotoxin contamination. The ODNs were reconstituted in saline for intraperitoneal injection in rats. For cell culture, the ODNs were reconstituted in PBS. Lipopolysaccharide from E. coli OIII:B4 was purchased from Invivogen (San Diego, CA; #LPS-EB). Rat macrophage colony stimulating factor (M-CSF) was from GenScript (Piscataway, NJ). All cytokine ELISA kits were purchased from R&D Systems (Minneapolis, MN). LDH kits were from Abcam (Cambridge, MA).

### Cell culture

BMDM were derived from both the femurs and tibias of SHR-SP rats as has been previously described, with slight modifications [42]. Bone marrow cells were cultured in DMEM supplemented with 10% FBS and 10 ng/ml rat M-CSF. 2x10^6^ cells in 11 ml culture medium were seeded in one 100 mm tissue culture dish. Three days later, 5.5 ml medium with 30 ng/ml rat M-CSF was added to each dish. On day 6, the cells were washed twice with PBS and scraped in the presence of cold HBSS. After centrifugation and resuspension, the cell density was adjusted to 2х10^5^ cells per ml and 2 ml cells were seeded in each well of six-well plates. Iba-1 staining indicated that >99% cells were macrophages. On day 7, the cells were treated with 1 ng/ml LPS and OGD with or without oligodeoxynucleotides for 18 hours. Supernatants were collected and used for both ELISA and LDH assays.

### Oxygen and glucose deprivation (OGD)

BMDM cells were seeded at a density of 2x10^5^/ml. After an overnight incubation, the cells were washed twice in PBS and 1 ml culture medium without glucose (Life Technologies, Grand Island, NY) was added to each well. The plates were placed inside modular incubator chambers (Billups-Rothenberg, Del Mar, CA) with anaerobic colorimetric indicator strips that detect a 0.2% oxygen threshold (Becton Dickinson). The chamber was flushed with a gas mixture of 95% N_2_ and 5% CO_2_ for 20 min at room temperature at 6 L/min. After flushing, the chambers were sealed and maintained at 37°C for 18 hours.

### Western blot

Total cell lysate was prepared via the use of a commercial lysis buffer (Thermo Fisher Scientific, Rockford, IL), which was added directly to each well of the six-well plates. After 15 min incubation on ice and 5s of sonication, the lysate was centrifuged at 10,000g for 15 min at 4°C. The supernatant was collected and the protein concentration determined via a BCA assay (Thermo Fisher Scientific, Rockford, IL). All samples were heated for 5 min at 95°C. 15μg of total cell lysate was used for each SDS-PAGE. The following primary antibodies were used for WB analyses: anti-IL-1β (R&D Systems, Minneapolis, MN; #AF-501-NA), anti-caspase-1 (Abcam, Cambridge, MA; #ab108362), anti-caspase 8 (Cell Signaling, Danver, MA; #4790), anti-NLRP3 (AdipoGen, San Diego, CA; #AG-20B-0014), anti-ASC (AdipoGen, San Diego, CA; #AG-25B-0006), anti-AIM2 (Santa Cruz Biotechnology, Santa Cruz, CA; #SC137967), anti-NLRP1 (Cell Signaling, Beverly, MA; #4990), anti-NLRC4 (Santa Cruz Biotechnology, Santa Cruz, CA; #SC49395), anti-iNOS (Abcam, Cambridge, MA; #ab3523), anti-caspase-11 (Santa Cruz Biotechnology, Santa Cruz, CA; #SC28230). Signals were detected using a chemiluminescent substrate − Immobilon Western (Millipore, Billerica, MA) followed by digital imaging with Fluor Chem camera (Alpha Innotech, San Leandro, CA) or C-Digit (LI-COR, Lincoln, NE).

The supernatant was concentrated using methanol/chloroform precipitation as has been described [43]. Briefly, 500ul supernatant was mixed with 500ul methanol and 125ul chloroform. After centrifuging at 16,000g for 5 min and removal of the top layer, 500ul methanol was added to the sample. Following centrifugation for 5 min at 16,000g, the pellet was then dried at 50°C for 5 min. The pellet was resuspended in 50 ul SDS loading buffer and heated at 95°C for 15 min.

### JC-1 assay

The MitoProbe JC-1 assay kit was obtained from Life Technologies (Grand Island, NY). BMDM cells were cultured in 100 mm dishes and subjected to 1 hour OGD in the presence of 1 ng/ml LPS + 50 ug/ml A151 or C151. After OGD, the cells were incubated with 2uM JC-1 in colorless DMEM medium at 37°C for 30 min. The cells were washed twice with cold PBS supplemented with 1.8 mM CaCl_2_, 0.8mM MgCl_2_, 10mM glucose, and 1 mg/ml BSA. The cells were scraped, centrifuged and resuspended in 500 ul colorless DMEM with 1uM DAPI for and prepped for FACS analysis.

### Stroke-prone spontaneously hypertensive (SHR-SP) rats

Male and female offspring (5–7 months of age) of SHR-SP breeders (a kind gift from Professor Yukio Yamori, Disease Model Cooperative Research Association, Hamamatsu, Shizuoka, Japan) were used throughout the course of this study. The National Institute of Neurological Disorders and Stroke Animal Care and Use Committee reviewed and approved all procedures. The rats were randomly divided into treatment groups; with each group containing between 7–18 rats. Each group received one intraperitoneal (i.p.) injection of oligodeoxynucleotides or saline 3 days before, 1 day before, or 3 hours after pMCAO. Oligodeoxynucleotide doses of 1 mg or 3 mg per rat were tested.

### Permanent middle cerebral artery occlusion (pMCAO)

Rats were anesthetized using 5% isoflurane for the induction phase and 2–2.5% isoflurane for the maintenance phase in a 30% O_2_ /70% N_2_O vehicle via a facemask. The rats were then placed in a lateral position, and a curved, vertical 2-cm skin incision was made in the midpoint between the left orbit and the external auditory canal. A 1 mm hole was drilled into the skull, 1 mm posterior and 6 mm lateral from the midline. A filamental probe was attached to the cerebral blood flow (CBF) monitor, followed by one small burr hole (1.5–2 mm) made via a high-speed microdrill through the outer surface of the skull at the junction between the medial wall and the roof of the inferotemporal fossa. The dura was opened with a 30-gauge needle to expose the middle cerebral artery (MCA), which was then occluded between the inferior cerebral vein and the lateral olfactory tract using a bipolar electrocoagulator. The coagulated MCA segment was then transected with microscissors to ensure that the occlusion was permanent. During pMCAO, the rectal temperature was monitored and maintained at 37±0.5°C with a heating pad (TCAT-2DF controller, Physitemp Instruments, Clifton, NJ); the rats were allowed to recover for 48 hours. 30% lidocaine was applied to the surgical sites to alleviate suffering during the post-surgical recovery. Of note, the surgeon was blinded to the treatment each of the rats received or was ultimately to receive. Upon sacrifice, the rats were asphyxiated via an overdose of isoflurane anesthesia followed by immediate decapitation.

### Neurological assessment

48 hours post-pMCAO, the rats were evaluated using methods that have been described previously [44]. However, we decided to modify the forepaw test to assess behavior in greater detail. Briefly, the animal was held by the base of the tail and allowed to grasp a horizontal bar. If both of the forepaws were able to grasp the bar immediately and at the same time, the grip-strength was defined as equal, score 0; if both of the forepaws grasped the bar immediately, and at the same time, but with decreased right forepaw grip-strength while the animal is gripping the bar as its tail is pulled up, a score of 0.5 was assigned; if both of the forepaws are able to grasp the bar, but with delayed motion and decreased strength of the right forepaw, a score of 1.0 was assigned; if the right forepaw was not able to grasp the bar, a score of 1.5 was assigned; if the rat displayed no motion/attempt to grasp the bar, a score of 2 was assigned. Of note, the scientist evaluating the neurological deficits was blinded to the treatment the rats had received.

### Infarct volume determination

Rats were euthanized 48 hours after pMCAO. The brains were removed, frozen on dry ice, and stored at −80°C for downstream analyses. Frozen brains were sectioned coronally at a thickness of 20-μm. One section was collected every 800-μm, with the discarded sections being used for RNA isolation and real-time (RT)-PCR. The cut sections were then fixed using paraformaldehyde vapor and stained with cresyl violet. Image J (NIH, Bethesda, MD) was used to quantify the infarct area. Infarct volume was calculated by summing cross-sectional areas and multiplying by the distance between sections, followed by a correction for brain swelling (edema) as has been previously described [45]. Again, the scientist calculating volume of infarction was blinded to the treatment of the rats.

### RNA isolation and RT-PCR

Frozen rat brain sections were homogenized in Qiazol lysis reagent (Qiagen, Valencia, CA). All of the reagents and primers used for RNA isolation and real time RT-PCR were purchased from Qiagen. Total RNA was isolated using the Qiagen miRNeasy mini kit according to the manufacturer’s instructions. RNA was then quantitated via a NanoDrop spectrophotometer (Thermo Fisher Scientific Inc). 1 μg RNA was used for cDNA synthesis using the QuantiTect Reverse Transcription kit. Real-time PCR was performed using a QuantiTect SYBR Green PCR kit and the following PCR primers: caspase-1 (#QT00191814), IL-1β (#QT00181657), NLRP3 (#QT01568448), NLRC4 (#QT01604652), Aim2 (#QT02376171), iNOS (#QT00178325), sdha (#QT00195958). The LightCycler 480 II (Roche, Indianapolis, IN) was used for reverse transcription and PCR. Melt-curve analysis was performed to affirm the single-band production. Gene expression was normalized to the housekeeping gene succinate dehydrogenase complex, subunit A (*sdha*) and the delta delta Ct method was used to compare amongst treatment groups.

### Statistical analysis

All data are expressed as mean ± standard error of the mean (SEM). All *in vitro* data were compared using the student’s t-test. Comparisons of infarct volume, and the neurological deficit score of two different treatment groups in rats were analyzed using the Mann-Whitney U test. Differences were considered significant when p < 0.05.

## Results

### A151 reduces the release of inflammatory factors and the death of BMDM induced by exposure to LPS and OGD

**Fig 1:**
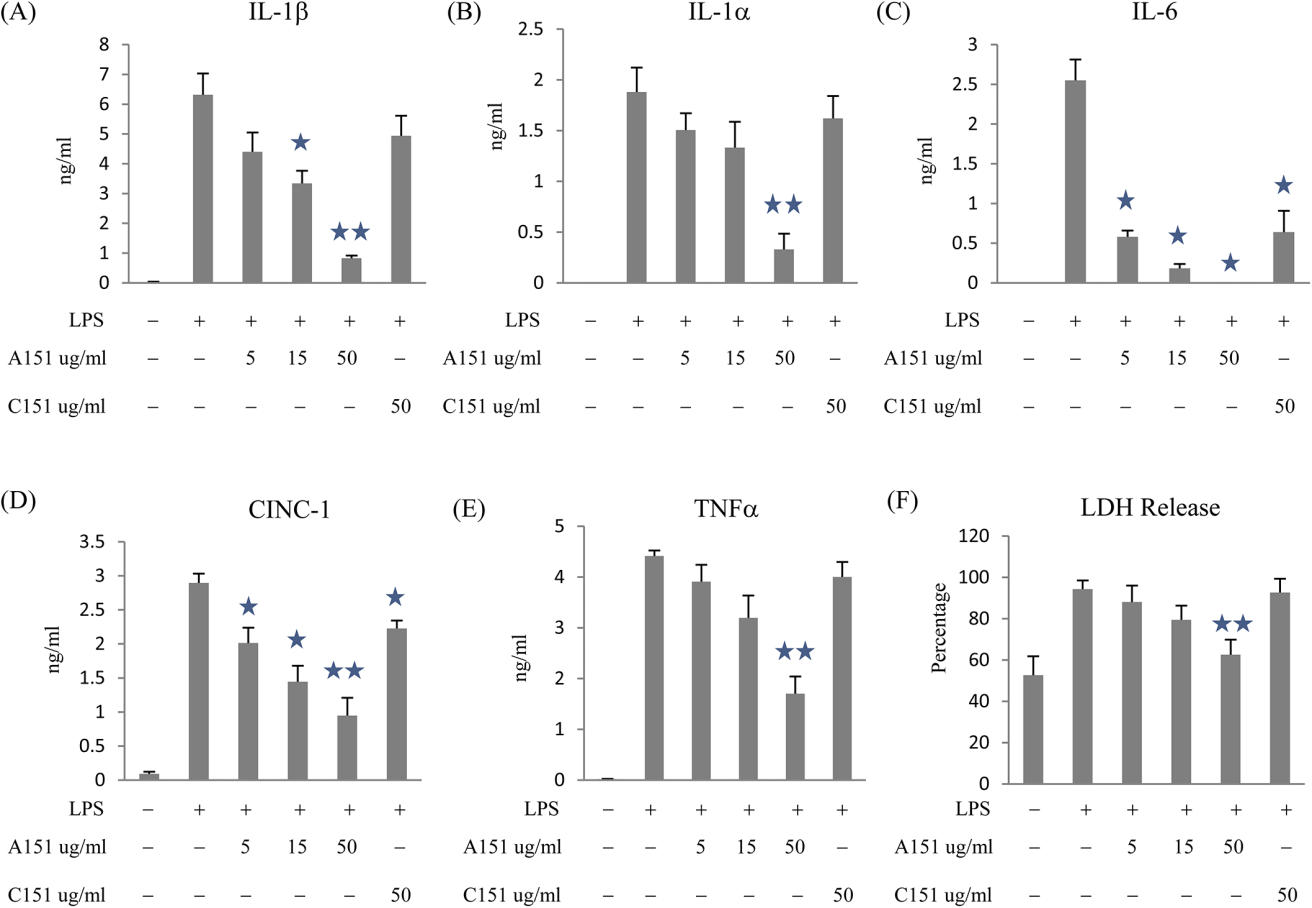
A151 reduced pro-inflammatory cytokine production and cell death in BMDM subjected to LPS and OGD. BMDM were treated with OGD, with or without 1 ng/ml LPS, A151 or C151 for 18 hours. IL-1β (A), IL-1α (B), IL-6 (C), CINC-1 (D), TNFα (E), and LDH (F) in cell culture supernatants were measured by ELISA. Data are presented as mean ± SEM from three replicates representative of three independent experiments (*, *p* < 0.05 compared with LPS treatment; **, *p* < 0.05 compared with control or C151 treatment).

Brain ischemia is characterized by oxygen and glucose deprivation and inflammation within the brain. To investigate the immunomodulatory potential of A151 under ischemic conditions, BMDM were treated with A151 or C151, LPS, and OGD. A151 dramatically reduced the levels of IL-1β, IL-1α, IL-6, CINC-1, and TNFα in culture supernatants (Fig 1). This was in contrast to the effects of the control ODN C151, which only reduced the levels of IL-6 and CINC-1. Of note, neither ODN altered the levels of CINC-3, IFNγ, IL-10, or TGFβ (data not shown).

Ischemia is also capable of inducing cell death, one form of which is pro-inflammatory cell death (i.e. pyroptosis). Because, pyroptosis has been associated with the release of pro-inflammatory cytokines from macrophages [15] and occurs in various forms of organ ischemia [46,47], we explored the effects of A151 on the survival of oxygen and glucose deprived BMDM. Compared with LPS treatment, A151 at 50 ug/ml reduced LDH release from 94.3 ± 4.2% to 62.6 ± 7.3% (p<0.05). Of note, C151 did not affect LDH release. These findings verify results from other groups, which have shown that the anti-inflammatory effects mediated by A151, are not due to an increase in target cell cytotoxicity [28,31].

### A151 reduces the maturation of IL-1β and caspase-1 and the expression of NLRP3 and iNOS in response to LPS and OGD stimulation

**Fig 2:**
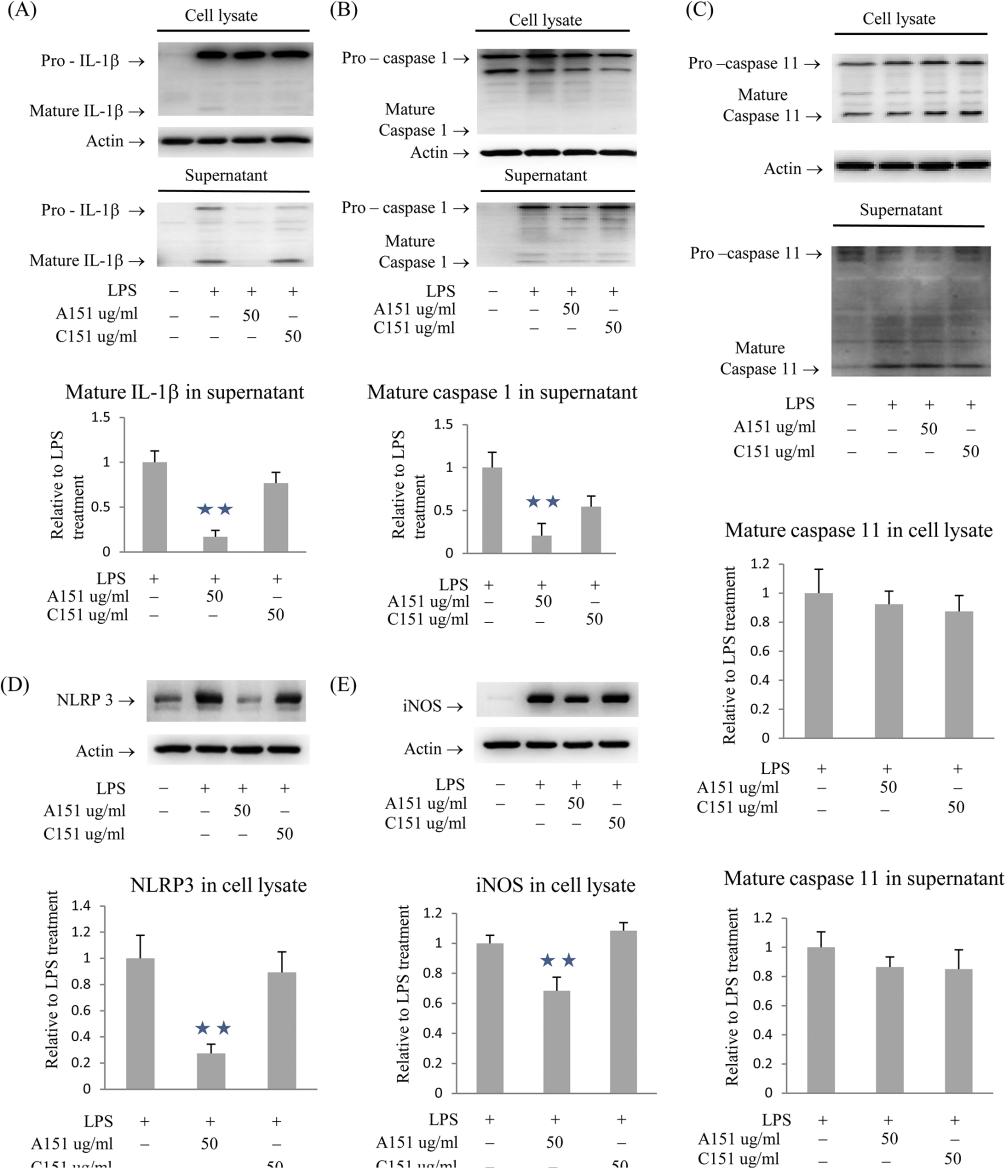
A151 reduced IL-1β and caspase 1 maturation, and the expression of NLRP3 and iNOS in BMDM subjected to LPS and OGD. (A) A151 reduced mature IL-1β in supernatants. (B) A151 reduced mature caspase 1 in supernatants. (C) A151 did not influence caspase 11 in cell lysates or supernatants. (D) A151 reduced NLRP3 in cell lysates. (E) A151 reduced iNOS in cell lysates. Data are presented as mean ± SEM from three replicates representative of three independent experiments (**, *p* < 0.05 compared with control or C151 treatment).

The effect of A151 on IL-1β expression and maturation was explored further by western blot analysis. A151 treatment of oxygen and glucose deprived BMDM reduced the levels of mature IL-1β in cell culture supernatants (Fig 2A). Being that the inflammasome is a multiprotein complex and a key regulator of IL-1β production, we studied the regulatory potential of A151 on the expression/protein levels of additional inflammasome components. A151 reduced mature caspase-1 (Fig 2B) and NLRP3 (Fig 2D), but did not affect ASC, AIM2, NLRP1 or NLRC4 (data not shown). Further it is known that IL-1β can induce the expression of iNOS [48] and that iNOS can influence stroke-induced cellular damage [49]. We therefore analyzed iNOS levels and found that A151 reduced the levels of iNOS expression (Fig 2E). It is important to note that in murine systems caspase-11 in part controls IL-1β secretion via the potentiation of caspase-1 activation and can thus induce caspase-1-independent pyroptosis downstream of non-canonical NLRP3 inflammasome activators. We thus sought to check the expression and maturation of caspase-11. Of note, A151 did not influence the levels of pro- and/or mature caspase-11 in cell lysates or supernatants (Fig 2C).

### A151 reduces the depolarization of mitochondrial membrane potential in BMDM

**Fig 3:**
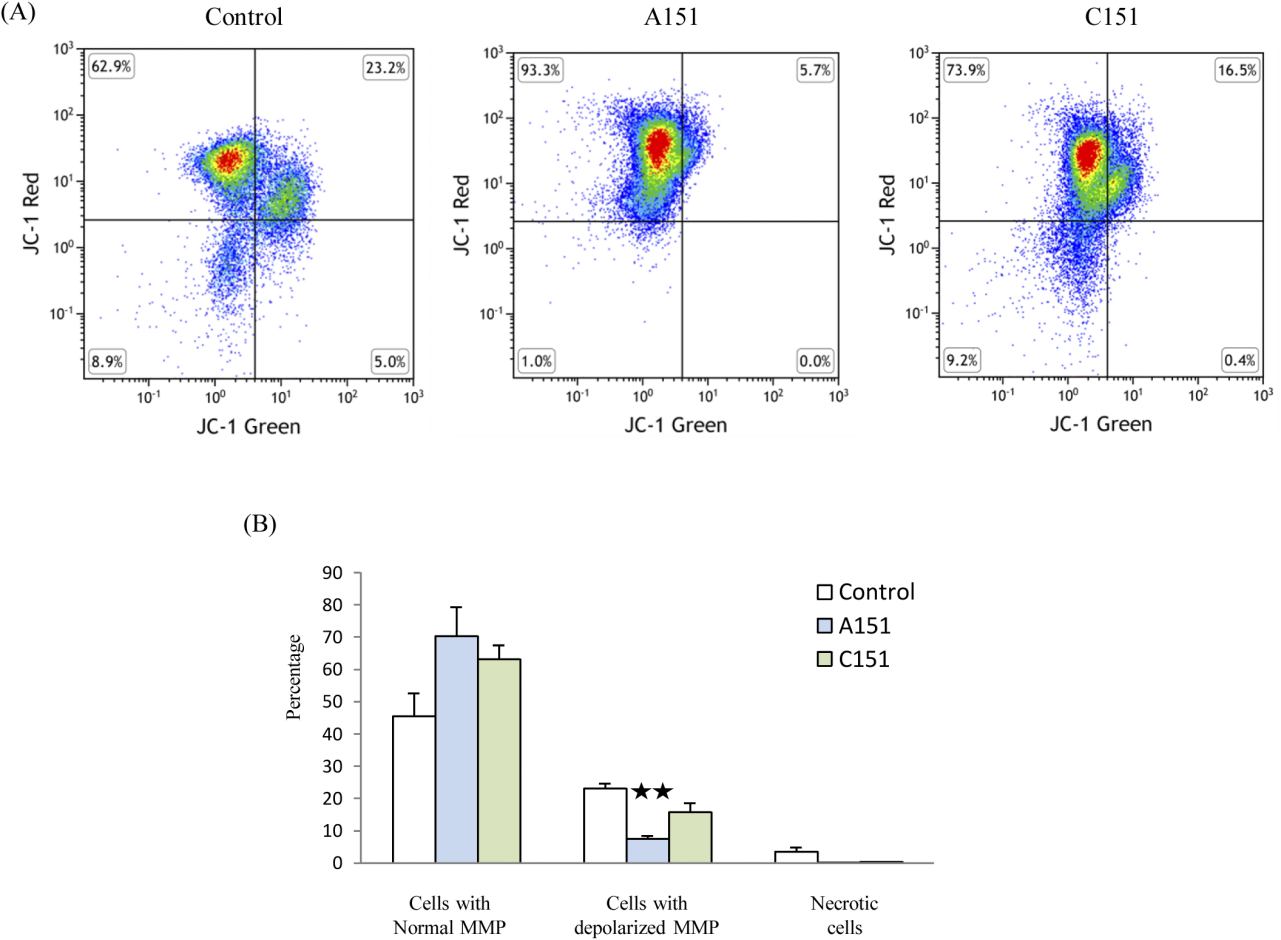
A151 reduced depolarization of mitochondrial membrane potential (MMP) in BMDM subjected to LPS and OGD. (A) FACS analysis of cells stained with JC-1. (B) The percentage of cells with depolarized MMP was reduced by A151 treatment. Data are presented as mean ± SEM from three replicates representative of three independent experiments (**, *p* < 0.05 compared with control or C151 treatment).

Mitochondrial dysfunction has been linked to NLRP3 inflammasome activation and we observed that A151 reduces NLRP3 protein expression [50]. We therefore sought to determine whether A151 could ameliorate BMDM mitochondrial dysfunction. To accomplish this we used the JC-1 assay to study the mitochondrial membrane potential (MMP) and found that A151 reduced the depolarization of MMP (Fig 3). Compared with C151 treatment, A151 reduced the percentage of cells with depolarized MMP from 15.8 ± 2.8% to 7.4 ± 0.9% (p<0.05).

### A151 reduces pMCAO induced ischemic brain damage in SHR-SP rats

**Fig 4:**
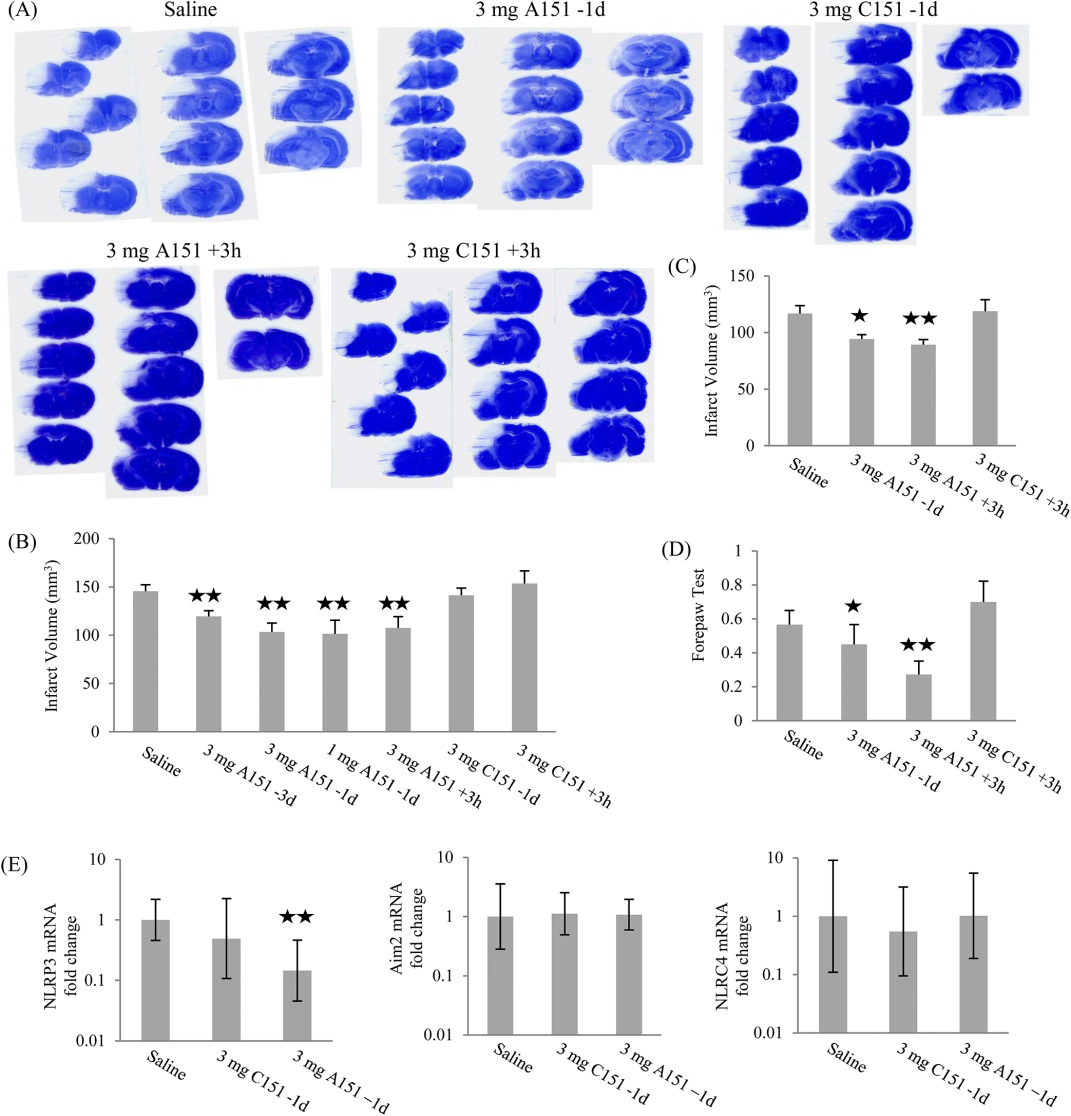
A151 reduced brain ischemic injury in SHR-SP rats 48 hours after pMCAO. The rats in saline groups were combined for analysis, as they were not statistically different. (A) Representative coronal brain sections stained with cresyl violet. (B) A151 reduced infarct volumes in male rats. (C) A151 reduced infarct volumes in female rats. (D) A151 improved performance in forepaw test in female rats. (E) A151 reduced brain NLRP3 mRNA 48 hours after pMCAO, the error bars represent the 95^th^ upper and lower confidence intervals of gene expression. (n = 7–17 per group; *, *p* < 0.05 compared with saline control; **, *p* < 0.05 compared with saline control or C151).

The ability of A151 to prevent and/or treat ischemic injury was evaluated in SHR-SP rats using the permanent middle cerebral artery occlusion (pMCAO) model. A single dose of 3 mg A151 was administered via i.p. injection 3 days prior to (-3d), 1 day prior to (-1d), or 3 hours post (+3h) pMCAO. Of note, we also tested 1 mg A151 administered 1 day prior to pMCAO in an effort demonstrate a threshold for effective dosing. Each of these treatment regimens significantly reduced infarct volumes (p<0.05, Fig 4). In male rats (Fig 4B), at 48 hours after MCAO, the infarct volumes (corrected for edema) of the saline treated (145.7 ± 6.6 mm^3^), 3 mg C151 -1d treated (141.3± 7.6 mm^3^) and +3h treated (151.2± 10.4 mm^3^) animals were similar; the infarct volumes in 3 mg A151 -3d treated animals (119.5 ± 5.8 mm^3^) averaged 15.4% smaller than in C151 -1d treated rats; infarct volume was decreased by 26.9% and 23.9%, respectively, in 3 mg A151 -1d group (103.2 ± 9.3 mm^3^) and +3h group (107.5 ± 11.7mm^3^); 1 mg A151 -1d reduced infarct volume (101.5 ± 14.0 mm^3^) by 28.1%. In female rats (Fig 4C), compared with the saline group (116.8 ± 7.1 mm^3^), infarct volume was decreased in 3 mg A151 -1d group (94.3 ± 3.9 mm^3^) and 3 mg A151 +3h group (89.2± 4.7mm^3^); 3 mg C151 +3h did not affect infarct volume (118.8 ± 10.3 mm^3^).

In addition to lesional volumes, neurological deficits were also evaluated at 48 hours after surgery. 3 mg of A151 improved the forepaw grasp and grip performance in female rats if administered 3 hours post-surgery (Fig 4D). Further, A151 (3 mg) -1d showed a trend toward improved forepaw performance. Unexpectedly in male rats, A151 did not improve forepaw grasp and grip performance. Further, we also tested the rats circling, swing, and side push responses, yet A151 did not affect these functions in either gender.

### A151 reduced NLRP3 mRNA within the brains of post-ischemic SHR-SP rats

We purified total RNA from the brain of SHR-SP rats 48 hours after pMCAO. All data were normalized against sdha mRNA levels and the expression of NLRP3 mRNA in the 3 mg A151 −1d rats were compared to rats treated with saline and 3 mg C151 −1d. Compared with saline and 3 mg C151 -1d treatments, the expression of NLRP3 was reduced by about 6.9-fold and 2-fold in 3 mg A151 −1d treated rats, respectively (p < 0.05, Fig 4E). Of note, the expression of Aim2 and NLRC4 were not altered by A151 (Fig 4E).

## Discussion

Inflammation has been shown to contribute to the development of strokes and to magnify subsequent brain damage [51,52]. Brain ischemia itself has been shown to trigger local inflammation that exacerbates brain damage and promotes stroke recurrence [8,53]. Thus, inflammation can influence both a patient’s prognosis and ultimate survival [54,55]. As such, the possibility that the temporally appropriate modulation of inflammation may be used to reduce ischemic brain injury is of interest.

This work provides evidence that A151, a synthetic oligodeoxynucleotide containing telemeric TTAGGG motifs, suppresses the production of inflammatory factors by BMDM subjected to OGD/LPS (e.g. CINC-1, IL-1α, mature IL-1β, IL-6, TNFα, and mature caspase-1) and reduces ischemic brain injury in SHR-SP rats that have undergone pMCAO. Having further explored the regulation of inflammasome sensors and adaptors, we noted that A151 reduces the expression of the NLRP3 protein in BMDM and NLRP3 mRNA within the ischemic brains of SHR-SP rats.

Inflammasomes act as sensors of both host-derived danger signals and infectious agents. Thus they play an important role in mediating inflammation in diseases including cancer, ischemia/reperfusion injuries, metabolic and autoimmune disorders [56,57]. Stroke involves an increase in extracellular ATP abundance, the production of reactive oxygen species, and necrotic cell death [8]. Critically all of these factors have been implicated in the activation of the NLRP3 inflammasome [58,59]. Beyond the induction of bioactive IL-1β, caspase-1 and NLRP3 also directly mediate cell death [60,61]. Immunoglobulin treatment, intermittent fasting, and intraperitoneal injection of the fungal isolate chrysophanol, all attenuate NLRP3 inflammasome activity and have been shown to reduce ischemic brain damage [24,25,62]. Our results thus add to the growing body of evidence indicating that ischemic stroke-induced brain damage can be ameliorated by modulating the NLRP3 inflammasome axis. Of note, the work of Denes et al. has suggested that both the AIM2 and NLRC4 inflammasomes (i.e. not the NLRP3 inflammasome) contribute to stroke pathogenesis [63]. While experimental models are often complicated by variations in animal species, age/gender, and anesthesia/surgical procedures it is prudent to note that Kastbom et al. have provided evidence linking genetic variants of NLRP3 with stroke in humans [64]. Clearly, the innate immune response after focal ischemia is complicated and further studies will be need to definitive clarify the core components involved and ultimately delineate species specific responses.

With the understanding that metabolic products have been shown to activate NRLP3 and mitochondria have been shown to be involved in NRLP3 inflammasome activation/function [65–67] we thought that the therapeutic effects mediated by A151 may proceed via this axis. Of note, recent work by Chang et. al. has directly linked the inhibition of the NRLP3 inflammasome to the preservation of mitochondrial integrity [68]. In an effort to determine if such a relationship does in fact exist within our experimental system a series of JC-1 assays were performed. This work has indicated that cells treated with A151 gave rise to both a greater proportion of BMDM cells in which mitochondrial membrane potential was intact and a decrease in those cells which displayed a decrease in mitochondrial membrane potential vs the control ODN, C151, after OGD. This result has clear implications and suggests a role for the mitochondria in A151’s suppression of inflammation. Further work will be necessary to dissect the precise roles of mitochondria ROS/DAMPs from the potential metabolic regulation of inflammation via A151 as recent evidence has come to suggest that mononuclear phagocyte (MP) polarization is accompanied by profound metabolic changes; pro-inflammatory MPs (M1) switch toward glycolysis, whereas anti-inflammatory MPs (M2) become more oxidative [69].

Our findings do not exclude the possibility that A151 may protect against brain ischemia by additional mechanisms. Multiple sensor molecules, including NLRP1, NLRP3, NLRP6, NLRP7, NLRP12, NLRC4, AIM2, and IFI16 can trigger inflammasome formation [12]. We have not excluded the possibility that A151 regulates the expression of inflammasome sensor molecules in addition to NLRP3 and/or influences the multimolecular assembly of the inflammasomes, however we have shown that the expression of Aim2 and NLRC4 were not altered by A151 *in vivo*. It is interesting to note that by supplementing extracellular poly(dA:dT) or by a DNA virus infection, Kaminski and colleagues showed that A151 binds to AIM2 and thereby competes for the binding of immune-stimulatory DNA [70]. Of interest, A151 has also been shown capable of promoting the generation of regulatory T cells which are protective in murine models of brain ischemia [71] [72]. Clearly, further investigation of the roles A151 is playing the regulation of other inflammasome components and in the assembly of inflammasomes is likely to reveal additional protective mechanisms pertinent for brain ischemia.

Though we have shown that A151 reduced pro-inflammatory cytokine production by activated macrophages, we do not exclude the possibility that A151 may protect the ischemic brain by changing peripheral immune responses. In line with such thinking it is prudent to note that remote ischemic conditioning (i.e. the application of brief episodes of ischemia and reperfusion to the remote organs/tissues) has been show to protect ischemic brain and heart [73]. Phosphorothioate ODNs administered by i.p. injection distribute systemically and reach most tissues including the bone marrow [74–76]. Monocytes, macrophages, lymphocytes, dendritic cells, and endothelial cells internalize and respond to phosphorothioate ODNs following parenteral administration [77–79]. Interestingly, A151 was shown to prevent the phosphorylation of STAT1 and STAT4 in LPS stimulated peritoneal macrophages [31], however the expression of phosphorylated STAT1 and STAT4 in BMDM subjected to OGD/LPS was not detectable in our experimental system.

Previous studies have examined the effects of administering immunostimulatory CpG ODNs in animal models of stroke. Briefly, CpG ODNs trigger TLR9 receptors and activate the innate immune system [80]. Systemic delivery of CpG ODNs prior to temporary MCAO reduced infarct volumes in mice and rhesus macaques via modulation of the TNFα axis [81,82]. Per the abovementioned findings we therefore conclude that A151 ameliorates ischemic brain injury through mechanisms that differ from CpG ODNs. Of note, the control ODN C151 reduced the production of IL-6 and CINC-1 in BMDM subjected to OGD and LPS treatment; this finding warrants the examination of sequence independent effects of suppressive ODNs in ischemic settings in the future.

Synthetic ODN A151 mimics the inhibitory activity of telemeric TTAGGG motifs and has been shown to slow or prevent the development of diseases characterized by excessive immune activation [83]. This raises concerns about the long-term administration of A151 which may reduce responsiveness to immunization and/or to infectious challenges [84]. However, available evidence suggests that A151 promotes the maturation of Th17 effector cells and improves host resistance to fungal pathogens [85]. The reduced infarct volume in the brains of SHR-SP rats with pMCAO demonstrated that a single administration of A151 is safe in an animal model of brain ischemia and in our studies we did not observe adverse effects on weight, serum chemistry, and/or blood composition (data not shown). In line with such findings it has been shown that administering A151 for up to 32 weeks does not adversely impact the health of mice [35].

The development of stroke and post-ischemic inflammation involves multiple cell types and inflammatory pathways. Blocking upstream components of inflammatory signaling and/or rationally targeting inflammatory pathways have shown efficacy in animal models of stroke [8]. Our study adds to this existing knowledge by demonstrating that oligodeoxynucleotides containing telemeric TTAGGG motifs reduce IL-1β and caspase-1 maturation, NLRP3 expression, and ischemic brain injury (i.e. lesional volume). Further investigation into the precise molecular mechanisms governing this immune modulation of post-stroke inflammation may lead to novel preventive and therapeutic strategies.
